# ‘More stressful than cancer’: Treatment Experiences Lived During Hurricane Maria among Breast and Colorectal Cancer Patients in Puerto Rico

**DOI:** 10.21203/rs.3.rs-2689228/v1

**Published:** 2023-04-03

**Authors:** Vivian Colón-López, Yara Sánchez-Cabrera, Marievelisse Soto-Salgado, Karen J. Ortiz-Ortiz, Troy Quast, María E. Fernández

**Affiliations:** Cancer Control and Population Sciences Division, University of Puerto Rico Comprehensive Cancer Center, San Juan; University of Puerto Rico/MD Anderson Cancer Center Partnership for Excellence in Cancer Research, San Juan; Cancer Control and Population Sciences Division, University of Puerto Rico Comprehensive Cancer Center, San Juan; Cancer Control and Population Sciences Division, University of Puerto Rico Comprehensive Cancer Center, San Juan; University of South Florida, College of Public Health, Tampa, FL; Center for Health Promotion and Prevention Research, School of Public Health, University of Texas Health Science Center, Houston, TX

**Keywords:** Cancer care during hurricane, breast cancer patients, colorectal cancer patients

## Abstract

**Background:**

This study explored experiences in cancer care and disruption after Hurricanes Irma and Maria’s aftermath in Puerto Rico (PR).

**Methods:**

A total of three focus groups were conducted among breast and colorectal cancer patients diagnosed six months before the disaster.

**Results:**

The most prevalent themes were (a) barriers related to their cancer treatment, (b) facilitators related to their cancer treatment, and (c) treatment experiences during the hurricane. Participants discussed struggles regarding their experience with treatment and access to care during and after Hurricanes Irma and Maria and how household limitations due to lack of electricity and water deter their intention to continue their treatment. Moreover, stressors directly linked with the disaster were the most challenging to cope with.

**Conclusions:**

Our study identifies the hardships experienced by cancer patients living during a disaster. Similarly, our study highlights the impending need to address in future emergency plans the individual and system needs of cancer patients in active treatment to minimize the delay in continuing cancer care.

## Background

The impact of disasters on public health has resulted in physical injury, acute disease, and emotional trauma, along with an increase in morbidity and mortality.^1^ Much of the research conducted in the aftermath of hurricanes and other natural disasters has focused on environmental risk factors, infectious diseases, physical hazards, and mental health. These studies have reported a higher risk of death, exacerbating chronic diseases, adverse mental health effects, substance abuse, among others.^2–8^ In September 2017, Hurricanes Irma and Maria devastated the Caribbean region, among them, the United States (US) territory of Puerto Rico (PR), with estimates between 800 and 8,500 excess deaths related to the hurricane through the end of December 2017, this added to massive resource and economics loss.^9^

Patients with chronic diseases like cancer have unique challenges and needs during and after disasters; with a greater risk of displacement, illness, or even death.^10^ Cancer patients are highly susceptible to disruptions caused by natural disasters, given that proper cancer treatment and follow-up involves communications with multiple healthcare providers^11^ that extend from diagnosis through treatment to survivorship.^12^ Additional challenges related to emergency preparedness planning often do not address cancer patients.^13^ Despite quantitative studies that have documented the impact of disasters on overall health and cancer, a qualitative approach has been less used to manage questions regarding the impact of the hurricane in care and to understand more deeply their views, opinions, and resilience stories from this experience. A previous study conducted in the University of Puerto Rico Medical Sciences Campus investigated the environmental stressors suffered by women with gynecological cancer after Hurricanes Maria and Irma in Puerto Rico and the Virgin Islands.^14^ Findings suggest that extreme heat as well as lack of access to essential services were the main concerns of women with gynecological cancers in PR.^14^ Similar to this, our study evaluates factors associated with cancer care disruption and time since the continuation of care in Hurricanes Irma and Maria’s aftermath among Hispanics living in PR focusing in two of the most incident cancers in PR, breast and colorectal cancer. To achieve this aim, we partnered with the Puerto Rico Central Cancer Registry (PRCCR), one of the oldest registries in America^13^, to develop a novel methodology in PR for breast and colorectal cancer patient recruitment in population-based research.

## Methods

We used a qualitative design through focus groups to accomplish the proposed objective. Focus groups can provide an in-depth understanding of factors influencing health behaviors by encouraging open discussion and increasing participants’ comfort level in disclosing personal opinions.^15^ We used a modified grounded theory approach for data collection and analysis.^16^ These models consist of systematic guidelines for collecting and analyzing qualitative data and comparing themes and emerging theories to data points.

The inclusion criteria for this study were the following: (1) patients diagnosed with breast (BC) or colorectal cancer (CRC), (2) aged 40 years and older being diagnosed between March 2017 and September 2017, (3) being under active cancer treatment before, during and after September 2017, and (4) lived and received treatment in PR during the aftermath of the events. We used data from the PRCCR to obtain a list of patients who met the inclusion criteria. A stratified random sample by geographical region was conducted to obtain 60 CRC and 60 BC patients that met the inclusion criteria.

**Figure 1:**
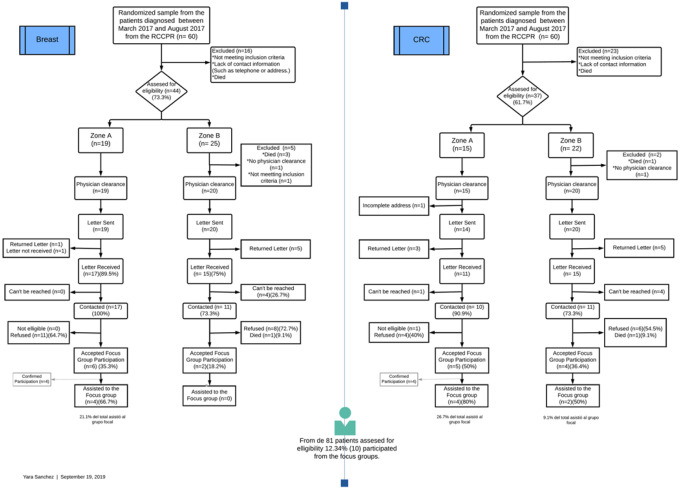
Case Ascertainment Protocol for Population Research Provided by the Puerto Rico Cancer Registry

Following the assessment of potential participants, our research staff followed the Case Ascertainment Protocol established by the PRCCR and developed in collaboration with the Principals Investigators (PIs) of this study (VC, KO) to contact potential participants (Fig. 1). Supplementary table 1 details the case ascertainment protocol selection of study participants from the random selection until completion of the focus group participation [see Supplementary Material file]. Like most central cancer registries in the US^17,18^, protocols establish that research staff cannot directly contact patients without consulting and receiving passive approval from their physicians. Therefore, we first got patients’ hematologist-oncologist and gave up to two weeks to report if a patient should not be contacted. After this passive approval, the research staff sent invitation letters by mail to the study participants. Information consists of an informed consent, a letter from the PIs and the PRCCR, as well as a brochure about frequently asked questions (FAQ) in cancer studies (e.g., *why members from this research study are contacting me?)*. The letter described the study’s objective and invited the participant to contribute to this study. In contrast, the PRCCR letter addressed the purpose of the registry and how they have their information. After two weeks of certified mail confirmation of receipt, we called the participants to discuss the study, assess their interest in participating in the study, and assess their eligibility. Research staff could contact potential participants up to a maximum of 8 times. A random sample of 120 patients was obtained from all the BC and CRC diagnoses between March 2017 and August 2017. After the case ascertainment protocol scrutiny, a total of 39 BC and 35 CRC cases were approved by their hematologists-oncologists to pursue contact and explanation of the research study. A total of eight BC and nine CRC cancer patients agreed to participate in the focus group. Three focus groups were conducted during May and June 2019. Two of the focus groups were conducted among CRC patients, and one of the focus groups was among BC patients. The number of participants in the focus groups ranged from 2 to 4. One of the participants was eliminated from the data analysis because we identified that the participant did not meet the inclusion criteria during the interview. The study protocol was approved by the Institutional Review Board of the UPRCCC (2018–09-03 B).

During the focus groups, the members of the research staff described the study objectives and obtained informed consent from all the study participants. All participants completed a short demographic questionnaire. Prior to the study commencement, the PIs were at the room, introduced themselves, assisted in the conduction of the short demographic questionnaire along with the staff, and discussed broad general themes. Focus groups were conducted in Spanish by the PIs of the study (VC, KO). Field notes were conducted during the focus group. Following the discussion, participants received educational materials about local organizations that offer services and support to cancer patients along with a $35 compensation. The focus groups lasted a total of two hours. Focus groups were audio-recorded, transcribed, and entered in *Atlas.ti* for analysis. Themes were derived from the data. Research staff analyzed all transcripts (VC, KO, MS, YS), created codes, and developed a codebook with operational definitions for each code created.

### Focus group guide.

The study team worked on a semi-structured interview guide (Table 1). Two research staff analyzed and coded the three transcripts. The most frequent codes were reviewed and summarized in the topics mentioned above.

## Results

Participants’ mean age was 61.8 years (standard deviation [SD], ± 10.87) at the time of cancer diagnosis. Most participants (80%) were married, and 40% of the focus group participants reported being retired or disabled at the time of the interview. Half (50%) of the participants reported a family household income below $15,000 annually, and 40% of them with education higher than high school. The two most common chronic comorbidities reported were: hypertension (80%) and diabetes (40%), in which all the participants who reported these conditions indicated they were diagnosed before the hurricanes. All participants started their cancer treatment before hurricane Maria, and 40% said that their treatment was interrupted (or delayed).

Analysis of the focus group identified (a) barriers related to their cancer treatment, (b) facilitators related to their cancer treatment and (c) treatment experiences during the hurricane, the most discussed themes.

### Barriers:

#### Basic needs and services.

Basic needs include an array of services such as: access to food, access to health services, access to shelter, among others. A lack of basic services has been identified as a common theme among participants of this study. The study participants commonly mentioned a lack of preparation at the time of the hurricane. Most of them were only prepared with canned food and water, some of them had a gas stove, and very few had electric generators or water cisterns.

“As for my condition well that caught us by surprise, because after the operation, remember that I got operated on September 8th. During Maria, I was discharged from [hospital] the same day that Maria came.”

– BC participant.

Study participants also recommended that PR becomes more prepared in terms of electricity, switching to renewable energy or other options, and for the health institutions in the island to get prepared with water cisterns and electric generators and the cities to have mitigation plans and collaborating committees. The participants recognized that there is little education on how to prepare for a hurricane and how to manage cancer patients during an emergency in PR. A participant mentioned: *“But there’s no type of education in terms of the condition”* — BC participant.

#### Health Facilities.

Health facilities services in PR were majorly disrupted due to the lack of electricity. The majority of the participants discussed how the hurricane delayed their schedules mastectomies and other treatment (e.g., chemotherapy and radiotherapy), which led to a postponement of their treatment by months. It was discussed by some participants how with the upcoming potential rescheduling of their cancer treatment, they still show hesitation to initiate the treatment due to their household situation (e.g., lack of electrical generator and lack of running water). Participants also expressed fear due to the instability of the electric system at the provider’s institution, which would affect their treatment. *“Radiotherapy was then scheduled for October. It was the partial mastectomy in August, then in Semptember, the ovaries [oophorectomy], and the radiotherapy in October. Everything was postponed. When [the institution] finnaly had electricity, they called me in October, and I said no, because I work in the metropolitan area and here half the island [shutdown] every day.”* — BC participant.

#### Communication and Coordination.

Difficulties reaching out to health care providers or facilities, as well as and lack of electricity where treatment took place, were among the most common codes identified. Participants mentioned the continuous calls and efforts they must conduct (particularly in rural areas); to reach for sufficient phone coverage and contact their providers. On the other hand, it was also discussed how they traveled to the metropolitan area to assess provider’s and treatment facilities’ availability or find out if drugstores were open. Participants mentioned how these barriers led to weeks (sometimes months) in determining and having plans regarding the next steps of their cancer care treatment. A participant discussed how during this waiting period, they relied on natural remedies since *“chemotherapy was not available, and my physicians did not have their facilities ready for treatment.”* — CRC participant.

#### Economic Availability.

Another barrier identified was economic availability, particularly the discontinuation of the special health coverage. In PR, the government provides special health coverage to patients with specific complex diseases, such as cancer. This special coverage aims to facilitate the management and treatment of these conditions among the population with the government health plan. Special coverage under this provision begins upon confirming a cancer diagnosis and ends after cancer treatment is completed. Some participants argue that the discontinuation of the special coverage after completing the chemotherapy affected follow-up services. *“I still haven’t done the PET scan because right after [Hurricane] María it was my appointment, […] I finished the chemotherapy, but right after the same day that the last chemotherapy was administered, they removed the service (health coverage) of the catastrophic”* — CRC participant.

“I looked for the referrals and even though, then, they called me about a month [later], this time it took a lot to get those referrals, [when] I delivered all the referrals and they told me, you no longer have the catastrophic [special coverage], you do not have the coverage, I had to pay for it and since I didn’t have the money, I didn’t do it, and it’s been a year and I haven’t done it yet.”

— CRC participant.

### Facilitators:

#### Social Support.

Other participants described how they did not have any major complications, qualifying their treatment experience during the hurricane as ‘normal’. One factor that might facilitate this experience was the social support encountered during the emergency, particularly in relation to the assistance in medications storage and transportation to the provider facilities: *“my sister had an electric generator, what she did was she took my insulin and put it in the fridge and I kept the bottle [insulin] I was using at the time.”*, other participant stated *“my daughter was staying at home, so there were 3 cars, there was gasoline. If I had to go get the [chemotherapy] my daughter would take me […], thank God.” —* CRC participants.

A group of participants expressed that their experience with cancer treatment during the hurricane was lessened due to their persistence and thrive *“Yes, in September was the hurricane, I had therapy the day before the hurricane and in October well I was taking my chemo again. Because I am persistent.” —* BR participant.

#### Service Availability.

Some participants identified some health services that were expedited during the emergency that helped alleviate their burden. On September 28th, 2017, the Puerto Rican government announced a waiver for pre-authorizations and medical referrals due to the crisis through an executive order (*Carta Normativa* CN-2017–221-D)^19^. This executive order stated that patients could get access to health services before the emergency required referral or pre-authorizations, which expedited the access to services during the disaster. In regards to this order, participants agreed this was a facilitator for their access to care. More so, due to their need to continuously visit their primary care physicians for requesting referrals for needed follow-up treatment, medications, or lab tests. A participant indicated:

“That [referral or pre-authorizations] should be removed. Leave it like that, that don’t require (referral) because you have to get a referral to go to a colonoscopy, you had to look for a referral for … and sometimes it was not easy, and with this hurricane, one went and there was no problem.”

— CRC Participant.

In addition, most participants expressed that access to their prescriptions during the hurricane was facilitated due to the opportunity to get their medications in advance (up to three months’ supply). In most cases, expedited communication with their providers and drug stores before the hurricane hits the island facilitates the rapid dispatch of medications.

### Treatment experiences during the hurricane:

#### Interruption and continuation of cancer treatments.

Participants discussed different experiences in the delay in their cancer treatment and how they were resilient and conformed with what was happening with their treatments. Some of the experiences were inherently due to the disaster and the inability to perform surgeries of other treatments *“I had surgery on January 20th, 2018. From there, I took 10 chemotherapies again and I am in treatment more or less.” —* CRC participant. Difficulties in coordinating the procedure for the supplementary device for the chemotherapy treatment (e.g., port-a-cath or chemo-port) was also mentioned. Other participant described interruptions in their cancer treatment due to clinical complications *“[…] On January 24th, I started chemotherapy, it was more or less 6 months that [then] turned into 9, because the moment came when the body could not resist the chemotherapy and instead of doing it every two weeks, I had to do it every three [weeks]. So, I came to finish almost in October or September 2018.” —* CRC participant. These complications were added difficulties in their treatment that were exacerbated due to the hurricane: *“In the middle of the hurricane, my body began to turn red, red, completely red and I didn’t know what to do, but luckily they explain [me what to do], that [the device] had a little key that closes, and I closed it. But I was completely red. Then the hurricane happened, one laying down all day and without disposition to do anything.” —* CRC participant. It was noted that despite these complications, overwhelmingly all the study participants did not consider transferring to other hospitals, institutions or to the US for continuation of their treatment as they express trust in their oncologists and medical team *“but I didn’t feel comfortable having to move farther, disarticulate the [established] medical team.”* —BC participant, this sentiment was mostly expressed due to their acceptance of the magnitude of the disaster and how it led to these delays and/or complications: “*No, because my chemo was every 3 weeks. Every 3 weeks I would take my chemo. It took a few days, but we have to understand that it was because of the hurricane. But I’m done, thank God.”* — BC participant

#### Hurricane-related stress.

The study participants discussed many stressors related to Hurricane Maria. A major stressor was the lack of water and electricity for a prolonged period. Due to the lack of electricity, participants discussed the need for being in long and extenuating lines to get gas for the generators; this experience was mentioned only for those participants who had this device at the time of the disaster. The economic burden that the patients overcome due to price increases in food and supplies was also mentioned. This was heightened by the limitation to use the Food Stamp Program card (cupones), as lack of communications impeded the use of the Electronic Benefit Transfer (EBT) cards system.

A surge in the prices of the generators resulted in additional financial strain:*”[I] bought a small electric generator, that now cost 3 or 4 hundred dollars, but at that moment (hurricane aftermath) we had to pay 2 thousand dollars, [I] only to use it to [keep] the refrigerator on.”* —CRC participant.

Not having electricity or a generator was more difficult for those who needed to keep their medications refrigerated (for example, insulin). Even the heat and mosquitos were stressful when not having at least a fan in their houses. The chores in the house can become overwhelming, and when is a working person with limited time is very difficult to get food and supplies for their homes to maintain their specific diets or bring food to their families. Moreover, overcoming this catastrophe been a cancer patient brought many limitations. Been recent through treatment, participants discussed the restrictions faced preparing for the hurricane. Study participants discussed hurdles in placing the shutters, making the long gas lines, going back to work, and completing the house chores.

When participants were asked about their reflections about the things most stressful about the hurricanes and their aftermath, participants reflected how the delay of treatment for their diagnosis was not stressful, as it was the stressors associated with the hurricane itself. *“I would say that, comparatively, the most stressful for me, but has nothing to do with me, the most stressful was seen other families without homes*…*” —* CRC participant. Similarly, when a participant was asked if the possibility of a delay in the treatment was stressful, she answered: *“No, because since I had a long time in conversation with the doctors, well I said, a bit more of waiting, because right now with the hurricane I can do nothing. That (delay of treatment) was not stressful.” —* BC participant.

## Discussion

This study assessed BC and CRC treatment experiences during and post-Hurricane Maria in PR. Given the high burden of chronic diseases in PR, most study participants had comorbidities in addition to their cancer diagnosis. Although most participants started their cancer treatment before the disaster, 40% indicated that their treatment was interrupted or delayed due to Hurricane Maria. Participants highlighted the island’s weak infrastructure system added to the stressors related to the disaster and the need to obtain basic household necessities as their main barriers. These issues affected the population at large and this study shows how impacted cancer patients in their initiation or continuation of cancer treatment. These barriers also limited patient’s confidence in the continuation of their treatment due to electric/water system frailty. These stressors were often mentioned as a more negative experience than the cancer diagnosis itself.

Difficulties in communicating with health care providers and facilities were undoubtedly one of the most pressing barriers during this disaster. This factor has also been acknowledged previously as one of the most influential service disruptions affecting the re-establishment of Public Health Laboratory activities in PR after Hurricane Maria.^20,21^ Despite being a primary barrier, participants assure us that moving to the US for the reinstatement of their treatment was not an option for them. However, it has been documented that this humanitarian assistance benefited other cancer patients on the island.^22^ Age and stage at diagnoses might influence the differences in the narratives observed in this focus group but reaffirm participant’s trust in Puertorricans physicians and their medical team despite the lack of communication and immediate follow-up of care caused by the hurricane.

It is important to highlight that despite the gradual reinstatement of health institutions, patients discussed their decision to delay their treatment. According to information from the PR Electric Public Authority, it took 11 months to restore PR’s power after Hurricane Maria.^23^ A shorter amount but still significant amount of time was recorded to reinstate water at the most rural part of the island. Social support at the household was limited due to the lack of fuel for transportation and accessibility barriers. Some neighborhoods limited visits to family members to provide care and support. Therefore, it is understandable the narratives of delay in initiating their treatment due to their household status. The effects of surgery and chemotherapy for either BC or CRC might be uncomfortable to manage without power (for ventilation, for example) and clean water (for cleaning their wounds and bathing facilities for dealing with vomit and diarrhea). This observation is important to emphasize as future estimates using the cancer registry, medical claims, and data conducted by our team might reflect a delay of treatment that was influenced by the state of the medical facilities on the island. In the context of Hurricane Maria and PR, these differences might vary by rural/urban neighborhoods.

Economic barriers, such as the cancelation of the catastrophic insurance (for patients insured with public health insurance) during their cancer treatment, exacerbated cancer patients’ challenges. However, this barrier is not directly related to an economic obstacle due to the hurricane. Nevertheless, it is still a major limitation among cancer patients who receive this special health coverage. On the other hand, follow-up and referrals for these patients were alleviated by an executive order in the aftermath of the hurricane that allowed patients of government healthcare to receive treatment and services without pre-authorization.^24^ In the context of studying the impact of Hurricane Maria on cancer, future studies need to explore how this policy facilitates (or deter) health services utilization (cancer screening, follow-up, referrals, treatment) as well as morbidity, mortality, and survival.

Moreover, throughout the focus groups, two main factors emerged from their narrative: their resilient nature and their faith in God. Resilience has been discussed in Hurricane Maria and cancer patients^25^ and historically documented among vulnerable populations. Studies on Hurricane Katrina^26,27^ have shown how low resilience and low social support were associated with avoidant coping in Hurricane Harvey^28^. It was also noted in our focus groups the role of family and religious beliefs as an essential component to their quality of life of wellbeing.^29^

## Conclusions

Barriers mentioned in our focus groups triggered a delay or postponement in treatment. Despite clinical institutions being open for service, factors related to the poor infrastructure of the island (electricity and water outage) and lack of communications limited their willingness to initiate or continue their cancer treatment. The experience of living in a disaster while recently diagnosed with cancer was more stressful than the diagnosis itself. This study has considerable strengths, including the use for the first time of the PRCCR as a tool for population-based research, with the development and implementation of a case ascertainment protocol for further research opportunities in the future. Opportunities to replicate a similar methodology in understanding cancer survivors or recently diagnosed cancer patients’ barriers will be pivotal in health care delivery research. Limitations of this study included the reduction of possible participants throughout the case ascertainment protocol, and the loss of participants that were receiving cancer treatment six months before the Hurricane and died between the hurricane and the recruitment process. Issues encountered were the lack of updated contact information of the potential participants from the PRCCR or the hematologist-oncologist, and the returned letters due to unclaimed or incorrect addresses. Despite these limitations, this effort will inform researchers and clinicians in factors affecting disruption and time to resume cancer care. Results from this study should inform disaster planners, emergency responders, and cancer care providers, in improving (or maintaining) policy. Our findings can also be used to develop disaster plans and educational materials for cancer patients after future natural disasters.

## Figures and Tables

**Table 1:** Interview Questions from Focus Groups Conducted in Puerto Rico with Breast and Colorectal Cancer Patients

Topic	Questions
Interruption and continuation of cancer treatments after hurricanes Irma and Maria	How many days after Hurricane Irma and Maria were you able to start or continue your cancer treatment?
Transportation Barriers	What challenges or barriers did you face in reaching the health organization or institution to receive your cancer treatment? Did you have someone to drive you to your medical appointments?
Communication and coordination of services with doctors and/or health facilities	What challenges or barriers did you face…? a. reschedule cancer treatments or other services after Hurricanes Irma and María? b. contact your doctor or any health institution/organization staff for information on the reestablishment of medical services? c. take or receive lab tests or results?
Physical and emotional stress during hurricanes Irma and María and its impact on the continuation of cancer treatment	Taking into consideration everything that happened with Hurricanes Irma and María, how stressful was your experience? Enter a value from 1 to 10, where 1 means no stress and 10 means a lot of stress.
	If you had to choose just one thing, what would be the most stressful thing about Hurricane Maria? Example: Floods, relocating temporarily due to losses at home, poor access to roads, lack of communication, electricity and/or water, limited access to gasoline, loss of your job, etc. Possibility of not receiving treatment for cancer or receiving it late?
	What do you consider to be the most stressful of handling your cancer condition during this period? *Example*: Concerned that your health would get worse if your treatment were delayed. Not having enough medications, not having contact with your doctor, not having means of transportation, changing clinic to continue treatment, etc. Avoid public places for fear of contracting infections that will worsen your health.

## Data Availability

All data analyzed during this study are included in this article and its supplementary information files.

## Abbreviations

BC: Breast cancer
CRC: Colorectal cancer
NCI: National Cancer Institute
PIs: Principal Investigators
PR: Puerto Rico
PRCCR: Puerto Rico Central Cancer Registry
UPR: University of Puerto Rico
US: United States
SD: Standard Deviation
